# Fractionation of sulfated galactan from the red alga *Botryocladia occidentalis* separates its anticoagulant and anti-SARS-CoV-2 properties

**DOI:** 10.1016/j.jbc.2022.101856

**Published:** 2022-03-23

**Authors:** Seon Beom Kim, Mary Zoepfl, Priyanka Samanta, Fuming Zhang, Ke Xia, Reena Thara, Robert J. Linhardt, Robert J. Doerksen, Michael A. McVoy, Vitor H. Pomin

**Affiliations:** 1 Department of BioMolecular Sciences, University of Mississippi, University, Mississippi, USA; 2 Department of Pediatrics, Virginia Commonwealth University, Richmond, Virginia, USA; 3 Center for Biotechnology and Interdisciplinary Studies, Rensselaer Polytechnic Institute, Troy, New York, USA; 4 Research Institute of Pharmaceutical Sciences, School of Pharmacy, University of Mississippi, Oxford, Mississippi, USA

**Keywords:** anticoagulation, oligosaccharide, sulfated galactan, surface plasmon resonance, viral inhibition, aPTT, activated partial thromboplastin time, AT, antithrombin, BoSG, *Botryocladia occidentalis* sulfated galactan, DMB, 1,9-dimethylmethylene blue, GFP, green fluorescent protein, hACE2, human angiotensin-converting enzyme 2, HCII, heparin cofactor II, HS, heparan sulfate, HSQC, heteronuclear single quantum coherence, MS, mass spectrometry, MW, molecular weight, RBD, receptor-binding domain, RU, resonance unit, S-protein, spike-protein, SA, streptavidin, SARS-CoV-2, severe acute respiratory syndrome coronavirus, SEC, size-exclusion chromatography, SPR, surface plasmon resonance, UFH, unfractionated heparin, VOC, variants of concern

## Abstract

Sulfation pattern and molecular weight (MW) play a key role in the biological actions of sulfated glycans. Besides anticoagulant effects, certain sulfated glycans can also exhibit anti-SARS-CoV-2 properties. To develop a more selective antiviral carbohydrate, an efficient strategy to separate these two actions is required. In this work, low MW fractions derived from the red alga *Botryocladia occidentalis* sulfated galactan (BoSG) were generated, structurally characterized, and tested for activity against SARS-CoV-2 and blood coagulation. The lowest MW fraction was found to be primarily composed of octasaccharides of monosulfated monosaccharides. Unlike heparin or native BoSG, we found that hydrolyzed BoSG products had weak anticoagulant activities as seen by aPTT and inhibitory assays using purified cofactors. In contrast, lower MW BoSG-derivatives retained anti-SARS-CoV-2 activity using SARS-CoV-2 spike (S)-protein pseudotyped lentivirus vector in HEK-293T-hACE2 cells monitored by GFP. Surface plasmon resonance confirmed that longer chains are necessary for BoSG to interact with coagulation cofactors but is not required for interactions with certain S-protein variants. We observed distinct affinities of BoSG derivatives for the S-proteins of different SARS-CoV-2 strains, including WT, N501Y (Alpha), K417T/E484K/N501Y (Gamma), and L542R (Delta) mutants, and stronger affinity for the N501Y-containing variants. Docking of the four possible monosulfated BoSG disaccharides in interactions with the N501Y mutant S-protein predicted potential binding poses of the BoSG constructs and favorable binding in close proximity to the 501Y residue. Our results demonstrate that depolymerization and fractionation of BoSG are an effective strategy to segregate its anticoagulant property from its anti-SARS-CoV-2 action.

A global pandemic caused by the severe acute respiratory syndrome coronavirus (SARS-CoV-2) or coronavirus disease-19 (Covid-19) was declared by the World Health Organization in March 2020 (1). The virus has rapidly spread to more than 222 countries since February 2020. SARS-CoV-2 is a zoonotic beta coronavirus primarily transmitted from person-to-person through respiratory droplets (2, 3). During the pandemic, global pharmaceutical companies started manufacturing vaccines, some of which have been approved by the US Food and Drug Administration under the Emergency Use Authorization. Vaccines have prevented some patients from becoming critically ill or dying and assisted in controlling the pandemic. Oral treatments of Covid-19, molnupiravir and paxlovid, have recently been issued an Emergency Use Authorization and are currently awaiting full approval by the FDA (4, 5). Nevertheless, discovery of new anti-SARS-CoV-2 agents is urgently needed, especially for treating infections by highly transmittable variants (6, 7).

The cell entry mechanism of SARS-CoV-2 has been well studied. The receptor-binding domain (RBD) in the S1 subunit of the spike-protein (S-protein) recognizes the heparan sulfate (HS) chains in cell surface proteoglycans and the human angiotensin-converting enzyme 2 (hACE2) (8, 9, 10, 11, 12). Structural analyses have revealed the mechanisms behind interactions between the SARS-CoV S-protein RBD and its host receptor hACE2 (13, 14). A heparin-binding site has been identified in the SARS-CoV-2 S-protein and the interaction with HS has been shown to be essential for hACE2 binding (12).

Since SARS-CoV-2 S-protein depends on cellular HS for cell binding, several recent studies have proposed anti-SARV-CoV-2 effects of exogenous sulfated glycans such as unfractionated heparin (UFH), nonanticoagulant heparin, and HS-based molecules derived from lung and other tissues in order to block virus binding (15, 16, 17). However, heparin and derivatives may present severe side effects or complications such as hemorrhage (18, 19), thrombocytopenia (20, 21), osteoporosis (22, 23), hypersensitivity reactions (24), and hypoaldosteronism (25). Hence, reports showing potential nonheparin anti-SARS-CoV-2 sulfated glycans, including unique molecules from marine sources, have recently appeared in the literature (26, 27, 28, 29, 30). However, these recent reports have studied the actions of the sulfated polysaccharides only against the Wuhan SARS-CoV-2 strain (WT) and not the mutated variants, including those of more infectious potency.

Viral mutation is naturally generated by multiple viral replications (31). RNA viruses are frequently and randomly mutated at higher frequency than DNA viruses because RNA polymerases lack exonuclease proofreading activity (32). Among all RNA viruses, coronaviruses mutate less frequently due to an enzyme that corrects errors during viral replications (32). Nonetheless, the number of variants of SARS-CoV-2 are increasing, mainly because of the pandemic. Variants have been identified worldwide, and a few such variants have been considered variants of concern (VOC) based on their harmful impact on human health. Such VOC may exhibit greater transmissibility, virulence, or immune escape mutations (33).

The N501Y mutation emerged among the Alpha variants and improves S-protein binding to cellular receptors and enhances virulence (https://www.gavi.org/vaccineswork/alpha-omicron-everything-you-need-know-about-coronavirus-variants-concern). Current SARS-CoV-2 VOC include Alpha (B.1.1.7), Beta (B.1.351), Gamma (P.1), Delta (B.1.617.2), and Omicron (B.1.1.529) (https://www.gavi.org/vaccineswork/alpha-omicron-everything-you-need-know-about-coronavirus-variants-concern). The N501Y mutation located in the RBD is found in all the VOCs except Delta. This N501Y mutation is associated with the highest transmissibility and has given rise to other lineages with additional mutations in the S-protein RBD (34). The emergence of a triple-mutated variant (K417T, E484K, N501Y, B.1.1.28), referred to as Gamma SARS-CoV-2, has been reported in Brazil (35, 36, 37, 38). The Gamma variant has shown high transmissible rates, inherent immune escape from neutralizing antibodies (39, 40, 41), and increased propensity of reinfection.

The main sulfated glycan isolated from the red alga *Botryocladia occidentalis*, a sulfated galactan (BoSG), has been well-characterized to possess not only antiviral activity (42, 43) but also antimalarial (44), anticoagulant (45, 46, 47, 48), and antithrombotic (46, 49, 50) activities. BoSG is structurally homogeneous in terms of its backbone, composed of the disaccharide repeating unit [→4)-α-_D_-Gal-(1→3)-β-_D_-Gal-(1→], but very heterogenous in terms of sulfation pattern (Fig. 1*A*). The anticoagulant and antithrombotic properties of BoSG have been widely studied and strong responses have been observed in these systems (51, 52, 53). Studies regarding mechanism of action have indicated activity toward heparin cofactor II (HCII) and antithrombin (AT), and dependency of molecular weight (MW) on these activities (47, 48, 49).

**Figure 1:**
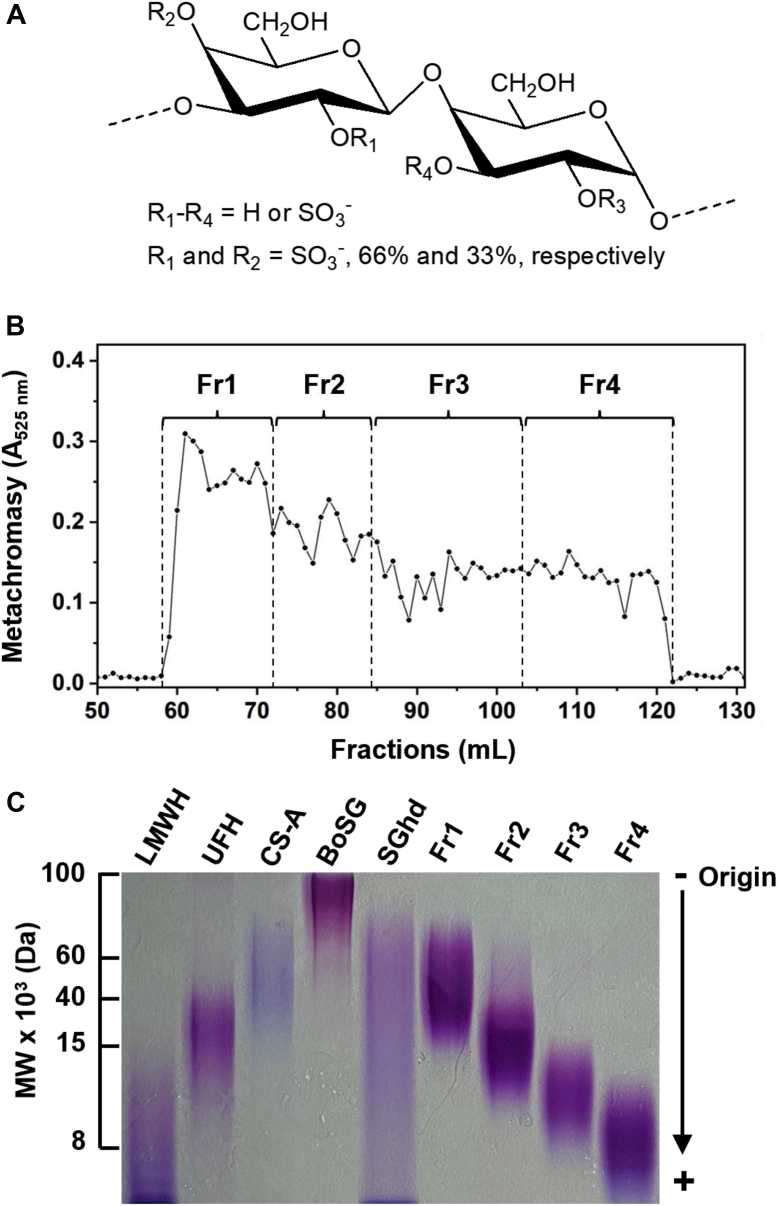
**Structure of BoSG, size fractionation, and MW estimation of low MW derivatives.***A*, BoSG is composed of a repeating disaccharide unit of the following structure [3-β-Gal2R_1_4R_2_-(1→4)-α-Gal2R3R-(1→]_n_, in which R = SO_3_^−^ or OH, R_1_ and R_2_ = 66% and 33% sulfation, respectively. *B*, oligosaccharides were produced by mild acid hydrolysis, fractionated by SEC using a Bio-Gel P-10 column, and detected by metachromasy using DMB (●). Fractions Fr1–Fr4 are indicated. *C*, the MW distribution of BoSG, hydrolyzed unfractionated BoSG (SGhd), and the four low MW fractions (Fr1–Fr4) were analyzed by PAGE along with the following molecular markers: low MW heparin (LMWH, ∼8 kDa), unfractionated heparin (UFH, ∼15 kDa), and chondroitin sulfate A (CS-A, ∼40 kDa). Samples (10 μg/each) were separated by 22% PAGE and stained with toluidine blue. BoSG, *Botryocladia occidentalis* sulfated galactan; MW, molecular weight; SEC, size-exclusion chromatography.

Here, we analyze the structural, anti-SARS-CoV-2, anticoagulant, and S-protein-binding properties of BoSG and its low MW derivatives. For this, a multifaceted approach was used through multiple analytical and biophysical techniques, including liquid-chromatography, electrophoresis, NMR, mass spectrometry (MS), surface plasmon resonance (SPR), computational docking, and assays measuring inhibitory activities against blood cofactors or cellular entry by SARS-CoV-2. Our results demonstrate that we can generate sulfated oligosaccharides from a marine alga with low residual of anticoagulant activity while retaining anti-SARS-CoV-2 activity.

## Results

### Purification of BoSG

Crude polysaccharides were obtained from the body wall of the red alga *B. occidentalis* through nonspecific proteolytic digestion using papain followed by ethanol precipitation, as previously described (48, 54, 55). The crude polysaccharides were subjected to anion-exchange chromatography on a DEAE Sephacel column and eluted with a linear NaCl gradient up to 3 M concentration (Fig. S1*A*). The fractions obtained by this chromatography were monitored by metachromasia using 1,9-dimethylmethylene blue (DMB) (56). The peak corresponding to BoSG started to elute at 0.9 M NaCl (Fig. S1*A*). After pooling the fractions belonging to this peak and desalination, the structural integrity of the BoSG preparation was confirmed by 1D ^1^H NMR (Fig. S1*B*), demonstrating a spectral pattern similar to previous work (48).

### Depolymerization of BoSG

Mild acid hydrolysis is routinely used for depolymerization of sulfated glycans, especially those for which specific digestive enzymes are unknown (57, 58, 59). The choice of 0.1 M hydrochloric acid and 60 °C temperature was based on previous reports (46, 49). To produce low MW derivatives from the above BoSG preparation that could be suitable for further fractionation by size-exclusion chromatography (SEC), different hydrolysis times (1, 3, 5, 7, 9, and 11 h) were evaluated. The MW distributions of the derivatives obtained were analyzed by PAGE (Fig. S2). Large-scale production of BoSG oligosaccharides was then made by 7 h of hydrolysis, since this time yielded a suitable MW range of medium-sized oligosaccharides to be filtered through the Bio-Gel P-10 column.

### Production of low MW BoSG derivatives

Approximately, 30 mg of BoSG was employed for production of oligosaccharides. The SEC column of choice was based on previous work (57, 58, 59, 60, 61). The Bio-Gel P-10 column has been reported to be effective for fractionation of medium- and/or small-sized sulfated oligosaccharides. Figure 1*B* shows the chromatographic profile of fractionation of BoSG derivatives monitored by metachromasy. Although no resolved peaks indicative of size-defined oligosaccharides were evident, four fractions (Fr1–Fr4) were randomly pooled. As analyzed by PAGE (Fig. 1*C*), oligosaccharides with different MW distributions were generated. The polydisperse nature of all four fractions indicated a complex and heterogeneous mixture of oligosaccharide chains within the samples.

### Structural analyses of the BoSG low MW derivatives

**Figure 2:**
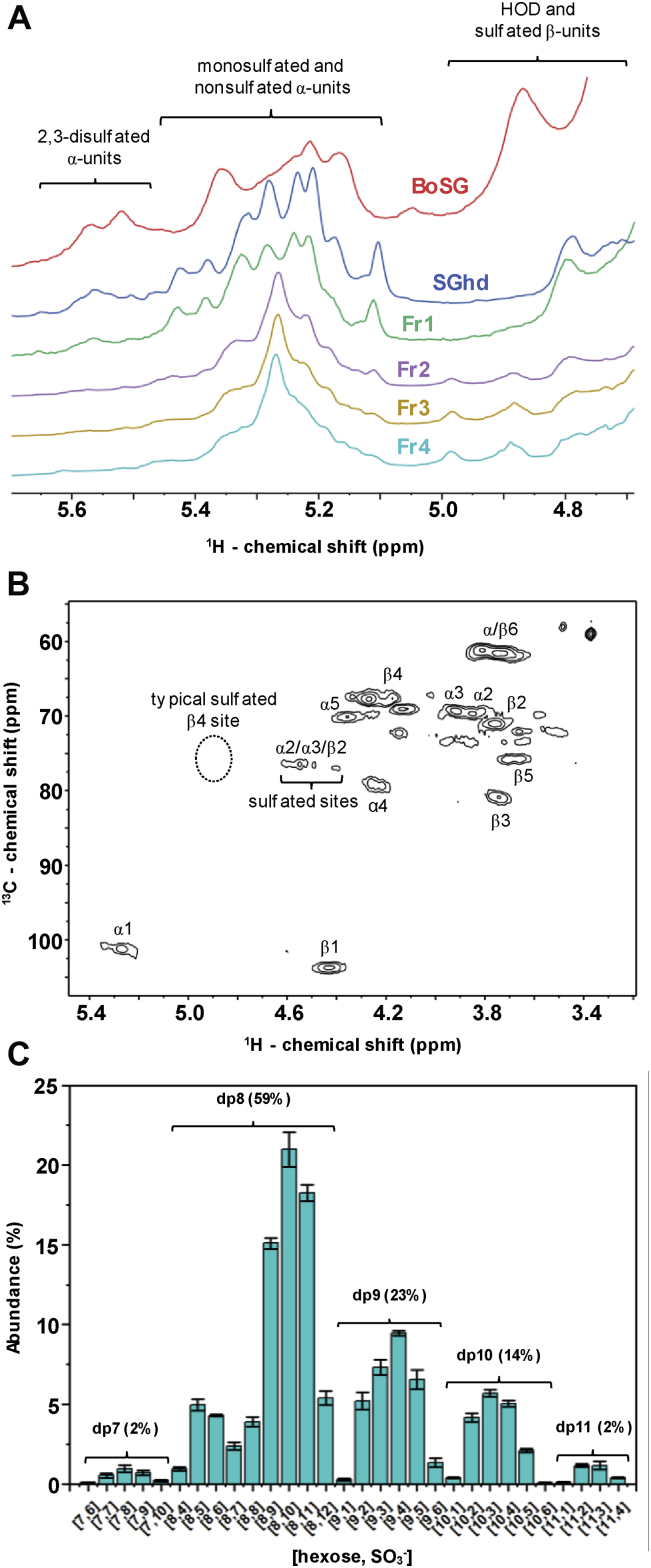
**1D**^**1**^**H NMR, 2D**^**1**^**H-**^**13**^**C HSQC, and top-down LC-MS analyses of BoSG fragments.***A*, 1D ^1^H NMR spectra of native BoSG (*red*), hydrolyzed unfractionated SGhd (*royal blue*), Fr1 (*green*), Fr2 (*purple*), Fr 3 (*yellow*), and Fr4 (*cyan*). *B*, 2D ^1^H-^13^C HSQC spectrum of Fr4 recorded in D_2_O at 50 °C on a 500 MHz Bruker NMR instrument. Chemical shifts are referenced to trimethylsilylpropionic acid to 0 ppm for ^1^H and methanol for ^13^C. ^1^H chemical shift ranges related to disulfated, monosulfated, and nonsulfated α and β units are indicated accordingly in the 1D ^1^H spectra in panel *A*. ^1^H-^13^C pairs of α and β units are labeled in the 2D cross-peaks of panel *B* using Greek letters denoting the anomeric (ring) unit followed by a number of the ^1^H-^13^C pair of the galactose ring. The typical region of the ^1^H4-^13^C4 cross-peak of a 4-sulfated β-galactose unit is indicated in the HSQC spectrum with *dashed circle*. *C*, percentage distribution of oligosaccharides of Fr4 according to their degrees of polymerization (dp) and sulfation number. BoSG, *Botryocladia occidentalis* sulfated galactan; HSQC, heteronuclear single quantum coherence.

NMR spectroscopy has been extensively used in structural characterization of BoSG (48). The 4-linked α-Gal unit can be 2,3-*O*-disulfated with an anomeric NMR ^1^H1 signal between 5.45 and 5.65 ppm, or 2-*O*-mono or 3-*O*-monosulfated with an anomeric NMR ^1^H1 signal between 5.10 and 5.45 ppm (Fig. 2*A* and Table 1) (48). The ^1^H1 chemical shift of the nonsulfated α-Gal unit of BoSG resonates within the same region as the ^1^H1 chemical shifts of the monosulfated region (Fig. 2*A* and Table 1). The ^1^H NMR spectra of the oligosaccharide fractions (Fr1–Fr4) clearly show a profile similar to the spectral profile of native BoSG. The major ^1^H1 peaks seen in the spectra of the fragments are clearly in the monosulfated and/or nonsulfated region of δ_H_ at 5.1 to 5.45 ppm. Hence, desulfation seems to occur during formation of the low MW derivatives produced by mild acid hydrolysis of BoSG. The characterization of the desulfation units was confirmed by the correlation of the ^1^H-^1^H COSY spectra (Fig. S3) and ^1^H-^13^C heteronuclear single quantum coherence (HSQC) (Fig. 2*B*) spectra of Fr4 compared with reference δ_H_ and δ_C_ values (62, 63).

**Table 1:** ^1^H and ^13^C chemical shifts of α- and β-galactose units with different linkages, anomericities, and sulfation patterns

Carbohydrate	Unit	^1^H and ^13^C chemical shift (ppm)^a^						Reference
		H1/C1	H2/C2	H3/C3	H4/C4	H5/C5	H6/C6	
Fr4	→4)-α-D-Gal-3(SO_3_^−^)-(1→	5.27/102.7	4.02/68.6	**4.61/77.8**	-	-	-	Current work
	→3)-β-D-Gal-2(SO_3_^−^)-(1→	4.44/105.3	**4.59/78.1**	*4.26/80.6*	-	-	-	Current work
BoSG	→4)-α-D-Gal-2,3(SO_4_)-(1→	5.62/100.5	**4.73/74.5**	**4.58/78.2**	-	-	-	Farias *et al.* (48)
	→4)-α-D-Gal-2(SO_4_)-(1→	5.27	4.59	4.05	-	-	-	Farias *et al.* (48)
Desulfated BoSG	→4)-α-D-Gal-(1→	5.29/102.9	3.85/71.5	3.95/70.9	*4.22/80.9*	4.16/73.9	3.78/63.0	Farias *et al.* (48)
	→3)-β-D-Gal-(1→	4.40/105.3	3.78/72.3	*3.75/82.7*	4.12/70.4	3.73/77.5	3.78/63.0	Farias *et al.* (48)
Sulfated galactan from *Gigartina skottsbergii*	→4)-α-D-Gal-2(SO_3_^−^)-(1→	5.45/94.7	**4.52/75.9**	4.11	-	-	-	Barahona *et al.* (84)
Sulfated galactan from *Styela plicata*	→4)-α-D-Gal-3(SO_3_^−^)-(1→	5.21/101.2	4.16/68.1	**4.65/76.9**	*4.51/77.4*	4.36/72.7	3.79/60.3	Albano *et al.* (62)
Sulfated galactan from *Glyptocidaris crenularis*	→3)-β-D-Gal-2(SO_3_^−^)-(1→	4.94/104.1	**4.52/80.2**	*4.11/81.8*	4.37/70.5	4.02/74.3	3.82/62.5	Castro *et al.* (63)
	→3)-β-D-Gal-(1→	4.73/107.2	3.87/72.0	*3.90/83.5*	4.24/69.1	3.75/77.0	3.82/62.5	Castro *et al.* (63)
Desulfated galactan from *G. crenularis*	→3)-β-D-Gal-(1→	4.79/103.5	3.90/69.9	*3.93/81.9*	4.29/68.3	3.82/74.8	3.87/60.5	Castro *et al.* (63)
Sulfated galactan from *Codium isthmocladum*	→3)-β-D-Gal-4(SO_3_^−^)-(1→	4.84/103.1	3.71/71.9	*4.16/77.6*	**4.94/77.8**	3.95/74.8	3.96,3.86/60.4	Farias *et al.* (85)
Desulfated galactan from *C. isthmocladum*	→3)-β-D-Gal-(1→	4.81/102.6	3.64/73.8	*3.92/82.9*	4.39/67.1	3.86/75.2	3.94,3.85/60.3	Farias *et al.* (85)
Sulfated galactan from *Meretrix petechialis*	→3)-β-D-Gal-2(SO_3_^−^)-(1→	4.83	**4.45**	*3.97*	-	-	-	Amornrut *et al.* (86)
Native galactan from *Echinometra lucunter*	→3)-α-D-Gal-2(SO_3_^−^)-(1→	5.47/97.2	**4.65/76.1**	*4.23/75.9*	4.35/73.9	NR/73.9	3.82/63.8	Alves *et al.* (87)
Desulfated galactan from *E. lucunter*	→3)-α-D-Gal-(1→	5.26/98.1	4.08/73.5	*4.14/77.2*	4.32/69.5	4.24/68.5	3.82/63.9	Alves *et al.* (87)

a Chemical shifts are referenced to external trimethylsilylpropionic acid at 0 ppm for ^1^H and methanol for ^13^C. Values in bold indicate sulfate position; those in italics indicate glycosylated positions.

The chemical shifts of sulfated units allowed identification of the 3-*O*-monosulfated α-Gal unit by the ^1^H1/^1^H2 (5.27/4.02 ppm) and ^1^H2/^1^H3 (4.02/4.61 ppm) ^1^H-^1^H COSY correlation as a [→4)-α-D-Gal-3(SO_3_^−^)-(1→] unit and of the 2-*O*-monosulfated β-Gal unit by the ^1^H1/^1^H2 (4.44/4.59 ppm) and ^1^H2/^1^H3 (4.59/4.26 ppm) ^1^H-^1^H correlation as a [→3)-β-D-Gal-2(SO_3_^−^)-(1→] unit (Fig. S3). Selective desulfation has already been observed from mild acid hydrolysis of other marine sulfated glycans (57, 58, 64) and should not be unexpected for the case of BoSG in this work.

Further analyses were conducted through a combination of 2D ^1^H-^13^C HSQC spectrum (Fig. 2*B*) and top-down liquid chromatography LC-MS (Fig. 2*C*) to assess more structural details of the low MW derivatives of BoSG after mild acid hydrolysis. Taking into account that Fr4 showed the most homogeneous 1D ^1^H NMR profile (Fig. 2*A*) and is also the fragment of the lowest MW distribution and potentially with the most structural modifications caused by the reaction, this fraction was chosen for these analyses.

From the assignments of the ^1^H-^13^C HSQC and ^1^H-^1^H COSY cross-peaks related to the sulfated sites and comparison of chemical shifts with references in the literature, we were able to clearly identify the presence of 3-*O*-sulfated α-Gal units and 2-*O*-sulfated β-Gal units in Fr4 (Fig. 2*B*). This can be seen from the cluster of ^1^H-^13^C HSQC and ^1^H-^1^H COSY cross-peaks related to monosulfated sites, δ_H_-δ_C_ from 4.40 to 74.5 to 4.73 to 80.2 ppm (Fig. 2*B* and Table 1). Note that there is clearly no ^1^H4-^13^C4 signal in the typical region of 4-sulfation for the β-Gal unit (δ_H_-δ_C_ at 4.94–77.8 ppm) in the HSQC spectrum (Fig. 2*B* and Table 1). This demonstrates the absence of 4-sulfation in the β-Gal units of Fr4. The ^1^H-^13^C HSQC spectrum also showed clear absence of the ^1^H1-^13^C1 cross-peak of the native 2,3-*O*-sulfated unit with typical δ_H_-δ_C_ at 5.62 to 100.5 ppm (Fig. 2*B* and Table 1).

The presence of only two sets of anomeric ^1^H-^13^C peaks, one with δ_H_-δ_C_ at 5.27 to 102.7 ppm belonging to the α-Gal unit and one with δ_H_-δ_C_ of 4.40 to 78.6 ppm belonging to the β-Gal unit (Fig. 2*B* and Table 1), as opposed to at least four anomeric HSQC cross-peaks seen in the native BoSG (47), indicates more homogeneous composition for Fr4 than for the native BoSG. From the NMR spectra (Fig. 2, *A* and *B*), this increased structural homogeneity in Fr4 can be attributed to the occasional desulfations that can occur on the disulfated Gal units during mild acid hydrolysis. The 1D ^1^H NMR spectra of fractions Fr2 and Fr3 (purple and yellow lines, respectively) as well as Fr4 (cyan line) in Figure 2*A* are very similar to each other, indicating similar homogeneity. In contrast, both the hydrolyzed unfractionated BoSG sample (royal blue line) and the fraction of highest MW (Fr1, green line) still showed some 2,3-*O*-disulfated α-Gal units, as in the native BoSG spectrum (in red). This can be seen from the presence of ^1^H peaks at the downfield region of the 1D spectra (Fig. 2*A*).

Figure 2*C* shows the percentage of the composing oligosaccharides in Fr4 obtained by the top-down LC-MS analysis. The bars indicate the oligosaccharide composition according to their number of hexoses along with their number of sulfate groups. More than half of the composing oligosaccharides in Fr4 have a degree of depolymerization (dp) equal to 8. This indicates a great portion of Fr4 as octasaccharide components. Two additional dp fractions also observed in Fr4 were dp9 and dp10. The major peaks of dp9 and dp10 (23% and 14% of the total content) show even lower numbers of sulfate groups/hexose units. This indicates the presence of monosulfated or nonsulfated units in dp9 and dp10. The numbers of sulfate groups in the bars with the highest abundance in the dp8 fraction showed 9 to 11 sulfates/8 monosaccharide units indicating therefore great abundance of monosulfated (and fewer disulfated) units in the dp8 fraction. These data corroborate well with the observation of the dominant presence of monosulfated units in Fr4 as seen through the 1D ^1^H (Fig. 2*A*) and 2D ^1^H-^13^C NMR spectra (Fig. 2*B*).

### Anticoagulant activities of BoSG and derivatives

**Figure 3:**
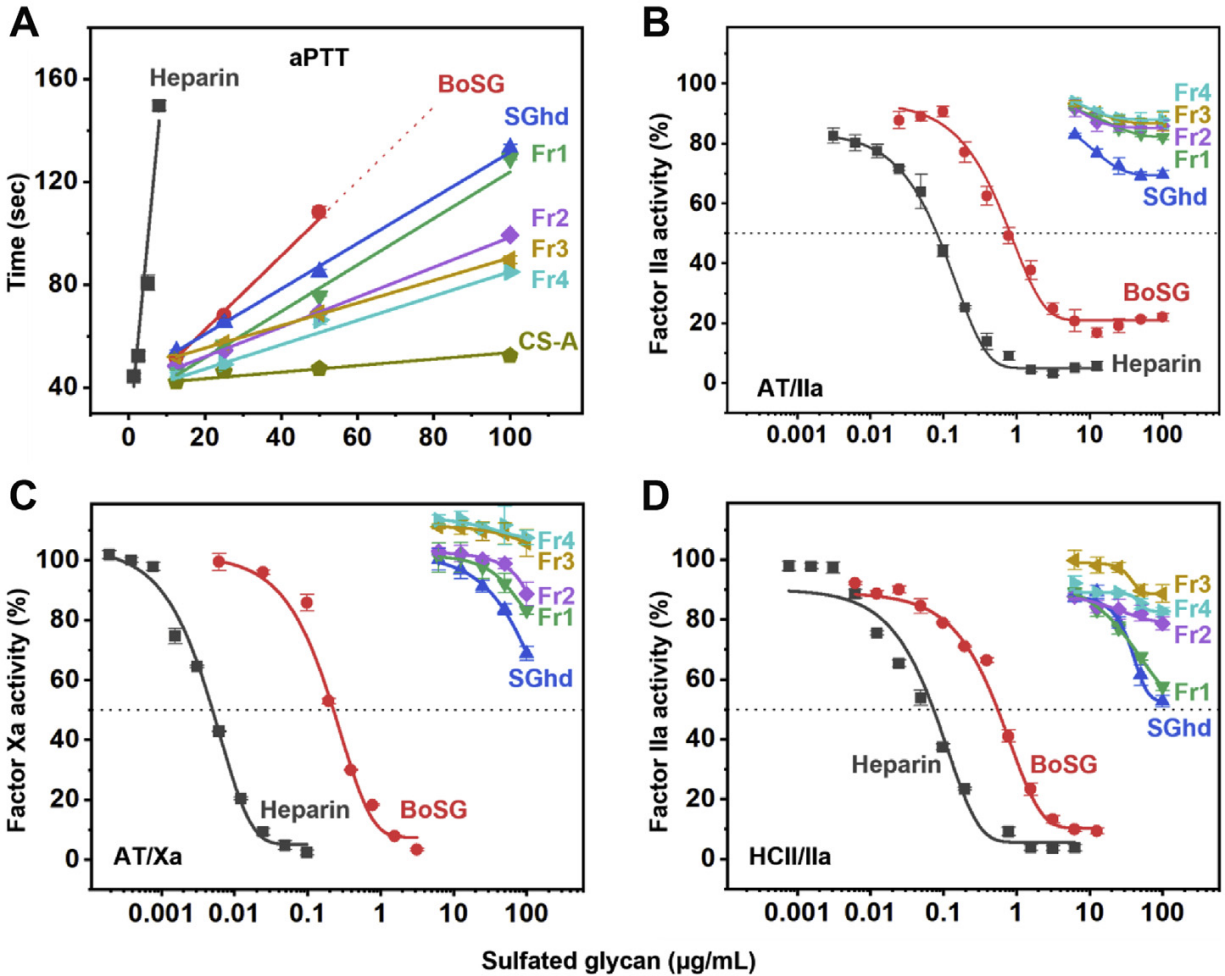
**Anticoagulant activity-concentration curves ofUFH,BoSG and derivatives.***A*, aPTT, (*B*) AT-mediated factor IIa inhibition, (*C*) AT-mediated factor Xa inhibition, and (*D*) HCII-mediated factor IIa inhibition. Sulfated glycans tested were unfractionated heparin (UFH, *black*), native BoSG (*red*), hydrolyzed unfractionated SGhd (*royal blue*), Fr1 (*green*), Fr2 (*purple*), Fr3 (*yellow*), and Fr4 (*cyan*). Concentrations of coagulation (co)factors were 10 nM of AT or HCII and 2 nM of factors IIa or Xa. aPTT, activated partial thromboplastin time; AT, antithrombin; BoSG, *Botryocladia occidentalis* sulfated galactan; HCII, heparin cofactor II.

The ideal anti-SARS-CoV-2 marine sulfated glycan would be devoid of anticoagulant effects (65). The native BoSG, a hydrolyzed unfractionated BoSG sample (SGhd,) and the four fractions of different MWs derived from BoSG (Fr1–Fr4) were subjected to *in vitro* anticoagulant assays to investigate the impact of MW on the anticoagulant action of BoSG. The methods employed were the activated partial thromboplastin time (aPTT) (Fig. 3*A*) and protease inhibition assays using purified serpins, AT (Fig. 3, *B* and *C*), and HCII (Fig. 3*D*), over the blood factors IIa (Fig. 3, *B* and *D*) and Xa (Fig. 3*C*). UFH with anticoagulant activity of 180 IU/mg was used in all experiments as a control. Anticoagulant activities of the six BoSG samples obtained from the aPTT curves (Fig. 3*A*) as well as the half-maximal inhibitory concentration (IC_50_) values obtained from the catalytic inhibitory curves (Fig. 3, *B*–*D*) are displayed in Table 2.

**Table 2:** *In vitro* anticoagulant properties of UFH, BoSG and derivatives through activated plasma thromboplastin time, inhibition of coagulation factors IIa and Xa by antithrombin, or heparin cofactor II in the presence of different sulfated glycans

Sulfated glycan	aPTT (IU/mg)^a^	AT/IIa	AT/Xa	HCII/IIa
		IC_50_ (μg/ml)		
UFH	180.00	0.08	0.0050	0.07
BoSG	21.09	0.82	0.23	0.55
SGhd	15.91	ND^b^	ND	ND
Fr1	13.19	ND	ND	ND
Fr2	9.80	ND	ND	ND
Fr3	7.54	ND	ND	ND
Fr4	8.64	ND	ND	ND

a Values were calculated using a parallel UFH (180 IU/mg) standard curve.

b Not determined.

From the data shown in Figure 3, anticoagulant properties can be seen for native BoSG (red curves), although it is significantly less active than UFH (black curves). BoSG is active in all anticoagulant systems. However, upon depolymerization, the anticoagulant properties of BoSG were significantly reduced. Loss of activity begins with the hydrolyzed unfractionated SGhd derivative (Fig. 3, royal blue curves). The fractionated derivatives with different MWs showed reduced activity as the MW decreases (Fig. 3 and Table 2). These results confirm that the anticoagulant action of BoSG is MW-dependent and the native MW is ideal for maximizing its anticoagulant action. Depolymerization of BoSG to produce lower MW fragments provides an efficient strategy to mitigate the anticoagulant properties of this marine carbohydrate.

### Anti-SARS-CoV-2 activities of BoSG and derivatives

**Figure 4:**
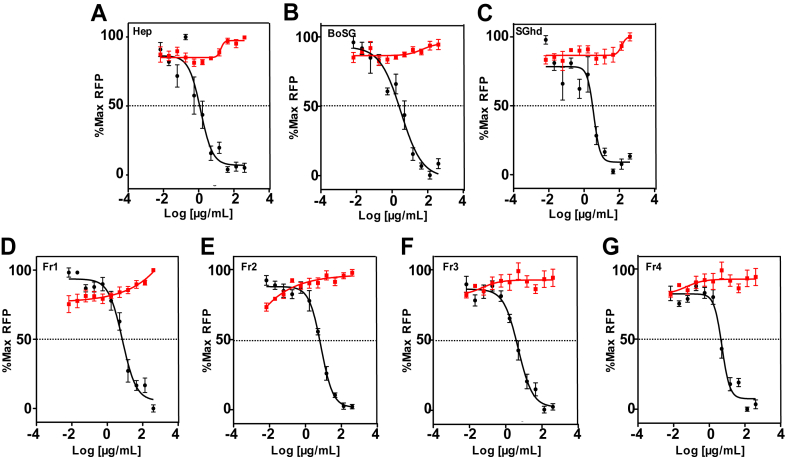
**Anti-SARS-CoV-2 activities ofUFH,BoSG and derivatives.** Confluent monolayers of HEK-293T-hACE2 cells in 384-well plates were treated with increasing concentrations of unfractionated heparin (*A*), native BoSG (*B*), hydrolyzed unfractionated SGhd (*C*), Fr1 (*D*), Fr2 (*E*), Fr3 (*F*), or Fr4 (*G*). After 1 h of incubation, cells were transduced with a lentivirus pseudotyped with SARS-CoV-2 (Wuhan strain) S-protein. Following incubation for 48 h, GFP fluorescence in each well was measured as RFU. Cell viability (*red*) was measured using the CellTiter-Glo assay as RLU produced from replicate uninfected HEK-293T-hACE2 cell cultures treated in parallel for 48 h. All values were normalized to % of maximum RFUs or RLUs. Data shown are means of three independent experiments ± SDs. BoSG, *Botryocladia occidentalis* sulfated galactan; RFU, relative fluorescence unit; RLU, relative light unit; S-protein, spike-protein; SARS-CoV-2, severe acute respiratory syndrome coronavirus.

The anti-SARS-CoV-2 activities of UFH, BoSG, and derivatives (SGhd and Fr1–Fr4) were determined by measuring reduction in green fluorescent protein (GFP) expression following transduction of hACE2-expressing HEK-293T cells with a lentivirus pseudotyped with SARS-CoV-2 S-protein (Wuhan strain) (Fig. 4). Half-maximal effective concentration (EC_50_) values obtained from the curves of GFP *versus* polysaccharide concentration are shown in Table 3. Complete inhibition could be achieved with all samples but in a concentration-dependent fashion (Fig. 4). Similar anti-SARS-CoV-2 activities were observed for BoSG and UFH, while anti-SARS-CoV-2 action of SGhd and Fr1–Fr4 were slightly lower than native BoSG (Table 3). All polysaccharides (UFH, BoSG, SGhd, and Fr1–Fr4) had no cytotoxicity at any tested concentration (red curves, Fig. 4). These results indicate that, although somewhat less active than native BoSG, low MW BoSG derivatives retain potent anti-SARS-CoV-2 activity.

**Table 3:** Inhibition of transduction by a lentivirus pseudotyped with SARS-CoV-2 S-protein

Sulfated glycan	EC_50_ (μg/ml)^a^
Heparin	2.11 ± 1.44
BoSG	2.00 ± 0.79
SGhd	3.55 ± 1.28
Fr1	6.59 ± 1.48
Fr2	6.34 ± 2.68
Fr3	6.29 ± 1.59
Fr4	5.51 ± 1.74

a Means ± SDs from three independent experiments.

### Binding properties of BoSG and derivatives with blood cofactors and S-proteins

**Figure 5:**
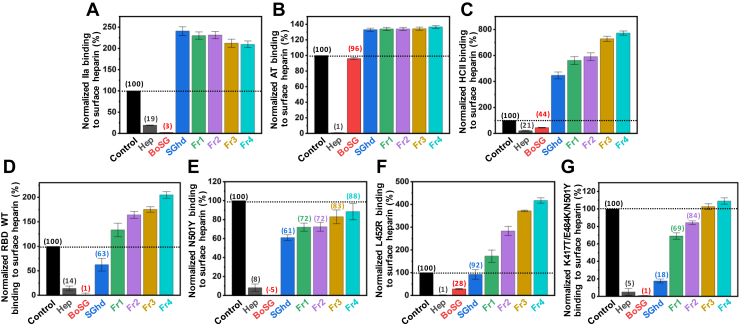
**SPR evaluation of protein–heparin binding and inhibition by BoSG and derivatives.** Bar graphs indicate normalized binding of IIa (*A*), AT (*B*), or HCII (*C*), or S-protein RBDs of WT Wuhan strain (*D*), or N501Y (*E*), L452R (*F*), or K417T/E484K/N501Y mutants (*G*) to surface-immobilized heparin. Inhibition of binding by sulfated glycans is shown for no-glycan control (*black*), UFH (*gray*), native BoSG (*red*), hydrolyzed unfractionated SGhd (*royal blue*), Fr1 (*green*), Fr2 (*purple*), Fr3 (*yellow*), and Fr4 (*cyan*). *Dashed lines* represent the control level in all panels. *Numbers* above bars indicate average inhibitory values. AT, antithrombin; BoSG, *Botryocladia occidentalis* sulfated galactan; HCII, heparin cofactor II; RBD, receptor-binding domain; S-protein, spike-protein; SPR, surface plasmon resonance; UFH, unfractionated heparin.

Competitive SPR using heparin immobilized on a streptavidin (SA)-coated sensor chip was used to investigate the binding of UFH, BoSG, and low MW derivatives with blood coagulation factors IIa (Fig. 5*A*), AT (Fig. 5*B*), and HCII (Fig. 5*C*), and four S-protein RBDs, including WT (Fig. 5*D*), Alpha N501Y (Fig. 5*E*), Delta L452R (Fig. 5*F*), and Gamma K417T/E484K/N501Y (Fig. 5*E*). Binding activities were normalized to the protein alone (black bars). Competitive IC_50_ values between the native BoSG and UFH are shown in Table 4. Based on these results, BoSG has strong affinity for IIa and HCII but low affinity for AT, as expected based on previous reports (48, 50). All low MW derivatives of BoSG (SGhd and Fr1–Fr4) showed no significant binding to the blood (co)-factors (Fig. 5, *A*–*C*). This observation is consistent with the MW-dependence of anticoagulant activity of BoSG derivatives seen in Figure 3.

**Table 4:** IC_50_ values for inhibition of binding by coagulation factors or S-proteins to immobilized heparin

Proteins	BoSG (μg/ml)^a^	Heparin (μg/ml)^a^
Thrombin	18.6 ± 0.7	41.6 ± 1.4
Antithrombin	ND^b^	14.6 ± 0.8
HCⅡ	83.4 ± 5.5	44.5 ± 4.5
RBD WT	9.0 ± 1.9	8.5 ± 1.7
RBD N501Y	4.4 ± 0.9	9.1 ± 1.4
RBD L452R	10.8 ± 4.0	6.1 ± 2.5
RBD K417T/E484K/N501Y	4.4 ± 0.8	7.7 ± 1.2

a Means ± SDs from three independent experiments.

b Not determined.

The binding of native BoSG (red bars) to the four S-protein RBDs was in general very similar to that observed for heparin (gray bars) (Fig. 5, *D*–*G*). This is consistent with the anti-SARS-CoV-2 data in Figure 4. Curiously, the BoSG low MW fragments showed very interesting and distinct binding properties among the RBD mutants. SGhd (royal blue bars) was able to interact with all RBDs but with different levels of affinity (Fig. 5, *D*–*G*). The best affinity was observed for the triple mutant (Fig. 5*G*). Fractions Fr1–Fr4 showed strong binding affinity for the Alpha N501Y mutant (Fig. 5*E*), some affinity for the Gamma K417T/E484K/N501Y mutant (Fig. 5*G*) and weak binding to WT (Fig. 5*D*) and the Delta L452R mutant (Fig. 5*F*). These results indicate that the BoSG derivatives have strong affinity for the RBDs that contain the N501Y mutation. In addition, through the competitive SPR assay (Table 4), it is also demonstrated that the native BoSG has stronger binding affinities for the N501Y-containing RBDs than for the single L452R mutant or WT RBD. The binding affinities of BoSG to the Alpha and Gamma variants (N501Y-containing strains) were approximately two-fold higher than those of UFH (Table 4).

### Molecular docking of BoSG-derived disaccharide constructs to N501Y RBD

**Figure 6:**
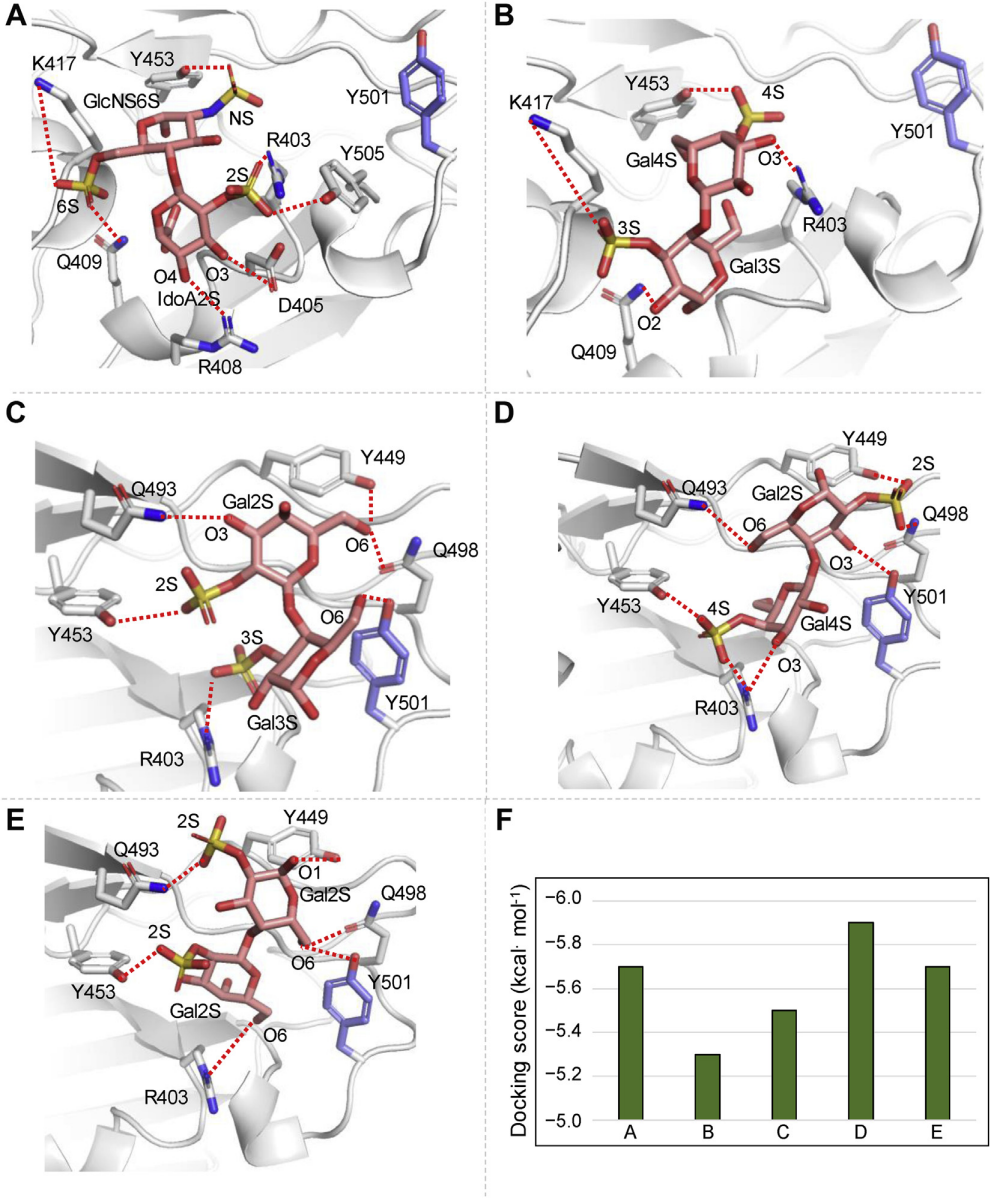
**Predicted binding poses of heparin and BoSG (monosulfated/monosaccharide) disaccharides bound to N501Y of SARS-CoV2 S-protein RBD.** Docked (*A*) heparin, (*B*) BoSG [4S-3S], (*C*) BoSG [2S-3S], (*D*) BoSG [4S-2S], and (*E*) BoSG [2S-2S] disaccharides in N501Y mutant. Selected neighboring interacting residues and the mutated residue are shown in *gray* and *purple*, respectively. *Dashed lines* indicate polar interactions between the RBD and glycan atoms. *F*, docking scores of the top-scored docked poses as obtained from AutoDock Vina. BoSG, *Botryocladia occidentalis* sulfated galactan; RBD, receptor-binding domain.

To define the contributions of different BoSG sulfation patterns to binding with the S-protein RBD, four possible BoSG (monosulfated/monosaccharide) disaccharides were constructed and computationally docked to the binding site close to the N501Y mutation in Alpha S-protein, which had the highest affinity observed by SPR. A heparin disaccharide was also constructed and docked for comparison. The binding site examined in this study was the same one previously used for the holothurian sulfated glycans in binding analyses to the WT and the N501Y mutant S-protein RBDs (65). The docking data are shown in Figure 6, and docking scores of the best-scored docked poses for the heparin disaccharide and the four BoSG disaccharides obtained from AutoDock Vina are shown in Figure 6*F*. The best-scoring docked pose of the heparin disaccharide to the N501Y mutant S-protein RBD indicated that its IdoA is oriented toward R408 and its O3 and O4 hydroxyl groups interact with D405 and R408, respectively (Fig. 6*A*). Heparin binding to the N501Y mutant lacked any interaction with Y501. A similar binding mode was observed for the BoSG [4S-3S] disaccharide (Fig. 6*B*). However, some residues such as D405, R408, and Y505 were not seen to be interacting with the BoSG [4S-3S] disaccharide, resulting in a slight worsening of the docking score as compared to that of the heparin disaccharide (Fig. 6*F*).

The other three BoSG disaccharides [2S-3S], [4S-2S], and [2S-2S], showed a similar overall binding mode (Fig. 6, *C*–*E*) but one that differs significantly from the heparin and BoSG [4S-3S] disaccharide binding modes. In the BoSG [2S-3S] disaccharide, Y501 forms a polar interaction with O6 of its reducing end Gal unit which contains the 3-sulfation (Fig. 6*C*). An electrostatic interaction between the 3-sulfation of the reducing end and R403 was additionally observed. Four residues, Y449, Y453, Q493, and Q498, formed polar interactions with O6, 2S, and O3 groups of the nonreducing end Gal unit of the BoG [2S-3S] disaccharide. Curiously, the Y453 residue showed significant interaction with one sulfate group of the nonreducing end Gal unit in each BoSG disaccharide (4S in Fig. 6, *B* and *D*; and 2S in Fig. 6, *C* and *E*).

In the BoSG [4S-2S] disaccharide, the 2S in the reducing end Gal unit forms two polar interactions, with Y449 and Q498 (Fig. 6*D*). The mutated residue, Y501, and Q493 form polar interactions with O3 and O6 of the reducing moiety, respectively. Similar to what was found for the BoSG [2S-3S] disaccharide (Fig. 6*C*), R403 formed an electrostatic interaction with the 4S group of the [4S-2S] disaccharide (Fig. 6*D*). An additional polar interaction was observed between R403 and the O3 group of the nonreducing end unit of this disaccharide.

Finally, for the BoSG homogeneously 2-sulfated disaccharide (Fig. 6*E*), the O1 and 2S groups of the reducing end unit formed polar interactions with residues Y449 and Q493, respectively. The O6 group of the reducing end showed polar interactions with Y501 and Q498. The nonreducing end of this BoSG disaccharide showed two key polar interactions of the 2S and O6 groups with residues Y453 and R403, respectively. For the two BoSG disaccharides containing 2-sulfation in the reducing end (Fig. 6, *D* and *E*), the overall binding orientation of the glycan was similar, with the subpocket-containing residue Y449 being occupied by the reducing end of the sugar. Overall, from the docking poses of the RBD–BoSG disaccharides, it could be seen that residues Y501, Y449, Q498, R403, Q493, and Y453 play key roles in binding with the S-protein RBD N501Y mutant.

## Discussion

Structural and functional investigations on the sulfated galactan from the red alga *B. occidentalis* (BoSG) are usually complex in nature. This polysaccharide, although highly potent in anticoagulation (48, 49, 50, 66, 67, 68, 69) and antithrombosis (46, 50, 70), can also show potential effects toward other systems (42, 44, 67), including virus-associated pathologies (42). In addition, the structure of BoSG is somewhat heterogeneous, not with regard to its highly homogeneous backbone (composed of repeating disaccharides with alternating 4-linked α-Gal and 3-linked β-Gal units), but in terms of sulfation patterns. Sulfation can occur at different sites of this molecule (C2 and C4 positions of the β-Gal unit and C2 and C3 positions of the α-Gal unit) and at different degrees (Fig. 1*A*). This makes structural investigations and consequent structure-activity relationships of BoSG laborious and complex.

We decided to follow here the strategy of depolymerizing BoSG for the production of oligosaccharides, to diminish the above-mentioned complexities regarding the structural and functional features of BoSG. Specific enzymes capable to cleave this marine polysaccharide (galactanases) are not widely known. Since chemical hydrolysis based on acidic digestive methods are well-established and commonly used for other marine sulfated glycans, such as sulfated fucans (57, 58, 59, 61, 71), and also previously reported for BoSG (49), we opted here to use mild acid for depolymerization of BoSG. The optimal conditions established were 0.1 M HCl at 60 °C for 7 h.

BoSG was properly depolymerized, giving rise to polydisperse oligosaccharide-based content with different MWs (SGhd, Fig. 1*C*). The MW of this digested material ranged roughly from 80 kDa to 2 KDa and size fractionation (Fig. 1*B*) led to a broad profile with no clear and defined peaks, indicating a lack of size- or structurally-defined oligosaccharide digestion products. This observation indicates nonspecific cleavage of glycosidic bonds of BoSG during the mild acid hydrolysis. In addition, desulfation specially at the C4 position of the β-Gal units was observed during the chemical depolymerization, as concluded from our analyses using 1D ^1^H, 2D ^1^H-^1^H COSY, ^1^H-^13^C NMR, and LC-MS (Fig. 2 and Fig. S3). Desulfation of BoSG during formation of low MW fragments by mild acid hydrolysis was not unexpected since selective desulfation (at the C2 position) has been reported during mild acid hydrolysis of other marine sulfated glycans (57, 58).

From the undefined SEC profile (Fig. 1*B*), four random fractions of BoSG oligosaccharides were pooled: Fr1 (MW ∼ 80–15 kDa), Fr2 (MW ∼ 40–10 kDa), Fr3 (MW ∼ 15–8 kDa), and Fr4 (MW ∼ 10–2 kDa), as roughly estimated based on PAGE (Fig. 1*C*). From the 1D ^1^H NMR spectra, the three lowest MW fragments (Fr2–Fr4) showed very similar spectra, in which the α-anomeric ^1^H region has a dominant peak of δ_H_ at 5.27 ppm (purple, yellow, and cyan lines in Fig. 2*A*). This signal was further characterized through the use of the ^1^H-^13^C HSQC and ^1^H-^1^H COSY spectrum of Fr4, indicating a monosulfated α-Gal unit. The ^1^H chemical shift of the ^1^H1 of all the 3-*O*-sulfated α-Gal units is very close in the spectrum (Table 1). The clear absence of the ^1^H4-^13^C4 signal of the 4-*O*-sulfated β-Gal unit was noted in the ^1^H-^13^C HSQC spectrum, indicating a lack of sulfation at the C4 of this 3-linked unit. Results from LC-MS (Fig. 2*C*) indicated three major conclusions: (i) dp8 (octasaccharide) is the major oligosaccharide component of Fr4; (ii) the formation of oligosaccharides with odd and even dp numbers indicates random cleavage of the glycosidic linkages of BoSG; and (iii) the major components of Fr4 are monosulfated/monosaccharides. From the set of structural results, we were able to demonstrate the structural heterogeneity of the BoSG fragments. For instance, Fr4 is composed of more than 20 different oligosaccharides (from dp7 to dp11, also with different sulfation contents per dp) (Fig. 2*C*). Further purification based on size and charge might be needed in future work concerning the actual development of a structurally defined BoSG-derived oligosaccharide as an anti-SARS-CoV-2 drug.

Previous reports have shown that BoSG is active in the coagulation cascade by enhancing the inhibitory activities of AT over factors IIa and Xa and HCII over factor IIa (47, 48, 49). Here, we investigated these anticoagulant systems using the BoSG low MW derivatives (Fig. 3 and Table 2). We observed a clear dependence on the MW of BoSG in anticoagulation. In our studies, only the native BoSG was capable of significant inhibition of coagulation activities in assays based on purified proteins (Fig. 3, *C* and *D*). The BoSG derivatives showed moderate activity in the aPTT system (Fig. 3*A*). Since these fragments were observed to be very inactive on the serpin-dependent mechanism (Fig. 3, *B*–*D*), the residual activity observed through aPTT is very likely due to the residual serpin-independent anticoagulant action of BoSG, as reported previously (45, 50, 68).

Although the residual anticoagulant action of low MW BoSG fragments is very weak, prophylactic monitoring of anticoagulant side effects should be carried out in case these fragments move on to clinical trials and use in human patients. The weak residual anticoagulant action of sulfated glycans used as antiviral therapeutics can be neutralized, if necessary, by administration of protamine sulfate, as performed for UFH. This is, however, much less likely to be necessary for the case of BoSG oligosaccharides because their residual anticoagulant activity is very low. It is known that LMWH does not change aPTT very much and does not present bleeding risk as a side effect. As opposed to UFH, which requires constant monitoring, LMWH-based treatment does not require monitoring. We hypothesize that the BoSG fragments may behave like LMWH given their low aPTT values. However, future *in vivo* studies must be made to confirm such hypothesis.

A previous report explained the reasons for the necessity of longer chains of BoSG to achieve the serpin-dependent anticoagulant action (49). Results have indicated that the template mechanism is more dominant than the allosteric mechanism in serpin-dependent thrombin inactivation and that longer BoSG chains are likely capable to hold multiple serpins (AT or HCII) and proteases (IIa or Xa) together. Decrease in the length of this polysaccharide has the indicated impact on its activity over the coagulation factors, with consequential reduction of the serpin-dependent anticoagulant action (49). Here, we demonstrate again that reduction in MW of BoSG decreases its anticoagulant effect, at least through the serpin-dependent mechanism.

In contrast, reduction of the MW of BoSG did not significantly impact anti-SARS-CoV-2 activity (Fig. 4 and Table 3). For instance, although ∼three-fold less active than native BoSG, Fr4, which is mostly composed of octasaccharides and generally monosulfated per monosaccharide, was very active in inhibiting the entry of SARS-CoV-2 S-protein-pseudotyped lentivirus, while SGhd and Fr1–Fr3 were only 1.5 and 3-fold, respectively, less active than native BoSG. This data suggests that a medium-sized BoSG oligosaccharide would be sufficient for inhibition of S-protein RBD interaction with HS or hACE2. Our SPR-based binding results have confirmed that reduction of MW of BoSG can significantly impact the anticoagulant outcome but not the anti-SARS-CoV-2 effect (Fig. 5).

One might question the impact of sulfation *versus* chain length on the biological systems, since besides MW reduction, we also observe desulfation during mild acid hydrolysis of BoSG. The distinct contributions of these two structural features (sulfation and MW) can be deduced by comparing the results obtained from PAGE (Fig. 1*C*), the 1D ^1^H NMR spectra of the BoSG derivatives (Fig. 2*A*), and the inhibitory curves (Figs. 3, *C* and *D* and 4). As can be seen, desulfation is dominant in Fr2–Fr4 since these derivatives showed the most modified 1D ^1^H NMR spectra than the native BoSG, especially regarding the changes on the downfield ^1^H signals of the 2,3-disulfated α-Gal units (Fig. 2*A*). Based on our analysis, disulfated α-Gal units are still significantly present in the SGhd (royal blue line in Fig. 2*A*) and Fr1 (green line in Fig. 2*A*). Hence, these fragments show chemical changes more with respect to MW than to sulfation (Fig. 1*C* *versus* Fig. 2*A*). The MW reduction from native BoSG (≥100 kDa, Fig. 1*C*) to the polydisperse MW distribution of SGhd (below 90 kDa) was, however, enough to significantly abolish the serpin-dependent anticoagulant property (red and royal blue curves in Fig. 3, *B*–*D* and Table 2) but not significantly impact anti-SARS-CoV-2 activity (Fig. 4, *B* and *C* and Table 3). This indicates a primary dependence on MW for the anticoagulation capability.

The anti-SARS-CoV-2 activities of SGhd and Fr1 (more sulfated molecules) as compared to Fr2–F4 (in which structural changes in these fractions are primarily MW reduction rather than desulfation, see Fig. 2*A*) were nearly equal (Fig. 4 and Table 3). This suggests little impact of MW or desulfation on anticoronaviral activity. In contrast, the anticoagulant effect of BoSG requires longer glycan chains to achieve the template serpin-dependent anticoagulant mechanism seen in the tertiary serpin–protease–glycan complex. In addition, longer BoSG chains can enable interaction and inactivation of multiple blood proteases by the same glycan chain. As opposed to the anticoagulant effect, SPR data (Fig. 5) indicates that inhibition of heparin binding by S-protein RBD containing the N501Y mutation can be accomplished just by medium-sized oligomeric lengths in order to achieve effective molecular interactions and consequent viral inhibition (Fig. 4).

Docking of the BoSG disaccharides to the N501Y mutant S-protein helped predict the overall poses of the BoSG derivatives in the binding site close to the 501Y residue and indicated the various possible pairwise mutant–BoSG polar interactions. This will identify all the possibly important S-protein RBD residues for potential modification to modulate glycan binding. Negative docking scores of the sulfated glycans indicated that binding to the site close to the 501Y residue was favorable.

Docking analysis (Fig. 6) has shown that heparin and the BoSG [4S-3S] disaccharide can interact with the S-protein N501Y RBD with similar binding poses between them (Fig. 6, *A* and *B*), but with different poses as compared with the three other BoSG disaccharides, which showed similar binding poses between them (Fig. 6, *C*–*E*). For the former two disaccharides, atomic interactions were observed between residues K417 and Q409 with the reducing end monosaccharide units (Fig. 6, *A* and *B*). For the latter three BoSG disaccharides, atomic interactions were observed between residues Y501, Y449, Q498, R403, Y453, and Y449 and the disaccharides. The studied BoSG disaccharides containing 2-sulfation at the 4-linked α-Gal unit at the reducing end ([2S-2S] and [4S-2S]) had the best docking scores although just slightly better than the other three studied disaccharides. In summary, sulfation and monosaccharide composition impact the binding properties of heparin and BoSG to the S-protein RBD sites near the 501Y residue.

### Conclusions

In this work, we were able to successfully produce a set of different oligosaccharide fractions from the structurally complex sulfated galactan from the red alga *B. occidentalis* (BoSG). Native BoSG shows both significant anticoagulant and anti-SARS-CoV-2 actions. The ideal anticoronaviral sulfated glycans would be those lacking activity on coagulation. This would make the polysaccharide more specific towards its antiviral action. Through a strategy of depolymerization, we were able to virtually abolish the anticoagulant action of the BoSG-derived oligosaccharides but still retain the anti-SARS-CoV-2 action. Here, we also noticed great selectivity of the BoSG and low MW derivatives toward the SARS-CoV-2 variants that express the N501Y mutation in their S-protein RBD. In conclusion, the absence of anticoagulant effects associated with specific anti-SARS-CoV-2 effects indicates the important and selective properties of the red algal sulfated glycan studied here.

## Experimental procedures

### Materials

Hydrochloric acid (# 320331, Sigma-Aldrich) was used for mild acid hydrolysis. Dialysis (# 9201735, Spectra Por) was used for desalting after isolation of BoSG through anion-exchange chromatography. Bio-Gel P-10 resin (# 150-4144, Bio-Rad Laboratories) and chromatography column (1.5 × 170 cm, # 7371598, Bio-Rad Laboratories) were employed for size fractionation of BoSG fragments. Sephadex G-15 resin (# G15120-10G, Sigma-Aldrich) and chromatography column (1.5 × 50 cm, # 7376607, Bio-Rad Laboratories) were used for desalting BoSG fragments. Chromatographic columns were connected to a peristaltic pump (# P-1, Pharmacia Fine Chemicals) to control the flow rate. Fractions from anion-exchange chromatography and SEC were collected using a fraction collector (# 2110, Bio-Rad Laboratories). After each chromatography samples were dried by lyophilization (# 7522900, Labconco). DMB was used for metachromatic assay. The power supply (# E0304) and electrophoresis kit (# 1658001, Bio-Rad Laboratories) were employed for PAGE. An ELISA reader (# SpectraMax ABS) was used to measure absorbance (525 nm) in the DMB and anticoagulant assays. SARS-CoV-2 S-protein RBD (wt), N501Y, L452R, and triple (K417T/E484K/N501Y) mutants were a gift from John Bates (University of Mississippi). Human AT, HCII, and α-Thrombin were purchased from Haematologic Technologies. Sensor SA chips were purchased from Cytiva. SPR measurements were performed on a BIAcore T200 operated using T200 control and T200 evaluation software (version 3.2).

### Extraction of BoSG

The marine red alga *B. occidentalis* was purchased at the Gulf Coast Ecosystems. *B. occidentalis* was lyophilized (# 7522900, Labconco) for 24 h, then suspended in 0.1 M sodium acetate (# S22040, RPI) buffer (pH 6.0) with papain (1 mg papain/g tissue) (# P4762, Sigma-Aldrich), 5 mM EDTA (# 03609, Sigma-Aldrich), and 5 mM cysteine (# C7352, Sigma-Aldrich), then incubated at 60 °C for 24 h. The incubation mixture was then filtrated and the supernatant saved. The resulting crude polysaccharide solution was precipitated by adding 16 ml of 10% cetylpyridinium chloride (# C0732, Sigma-Aldrich) solution and allowed to stand at room temperature for 24 h. Following centrifugation at 3000 rpm for 30 min, the pellet was washed with 610 ml of 5% cetylpyridinium chloride solution, centrifuged again, then dissolved in 172 ml of 2 M NaCl (# S23020, RPI) containing 15% ethanol (v/v) (# 2701, Decon Lab Inc) and precipitated by adding 305 ml of absolute ethanol followed by incubation at −20 °C for 24 h. The sample was centrifuged at 3000 rpm, washed twice with 305 ml of 80% ethanol, and washed again with 305 ml of absolute ethanol. The final precipitate was dried at 60 °C overnight to produce the crude polysaccharide.

### Purification of BoSG

The crude polysaccharide was subjected to anion-exchange chromatography using a DEAE Sephacel resin (# 17-0500-01, Cytiva) column (2.5 × 20 cm, # Bio-Rad Laboratories) equilibrated with 50 mM sodium acetate. Fifty millimolar sodium acetate with 3 M NaCl was used as second mobile phase to generate a salt gradient. The gradient produced by a gradient mixer (# GM-1, Pharmacia Fine Chemicals) was applied over 24 h and ranged from 0 to 3 M NaCl in 50 mM sodium acetate. The flow rate was set to 3.5 ml/10 min/fraction collected in the fraction collector (# 2110, Bio-Rad Laboratories). Fractions of different anionic content were monitored by DMB (# Sigma-Aldrich) (Fig. S1*A*).

### Depolymerization of BoSG

Mild acid hydrolysis conditions were optimized by incubating a 1.0 mg/ml solution of BoSG in 0.1 M HCl at 60 °C for 1, 3, 5, 7, 9, or 11 h. Aliquots of 1.0 ml were collected at each time and neutralized by adding an equal volume of ice-cold 0.1 M NaOH. Hydrolyzed products were analyzed by PAGE (12%) to determine MW distributions of each sample (Fig. S2). The 7 h hydrolysis time was selected producing a suitable MW distribution. The process was scaled up for preparation of 30.0 mg BoSG dissolved in 6.0 ml of 0.1 M HCl at 60 °C for 7 h, and the pH was then adjusted to 7.0 by addition of 6.0 ml 0.1 M NaOH. Hydrolyzed products were then lyophilized.

### Fractionation of BoSG fragments

Hydrolyzed products were fractionated by SEC on a Bio-Gel P-10 column (1.0 ml/15 min/fraction). The resin was equilibrated with aqueous 10% ethanol containing 1.0 M NaCl as described previously (61, 72). The collected fractions were assayed for metachromatic properties using DMB (73). Four major fractions (Fr1–Fr4) containing various heterogeneous oligosaccharides were lyophilized, dissolved in 1.0 ml of distilled H_2_O, and desalted using a Sephadex G-15 column (1 × 30 cm) eluted with distilled H_2_O. MW distribution of fraction was determined by PAGE (22%) using the MW standards LMWH (∼8 kDa), UFH (∼15 kDa), and CS A (∼40 kDa) (Fig. 1*C*). PAGE gels were stained using 0.1% (w/v) toluene blue in 1% acetic acid (v/v) and destained with 1% acetic acid.

### NMR spectroscopy

1D (^1^H) and 2D (^1^H-^13^C HSQC, ^1^H-^1^H COSY) spectra were recorded using a Bruker Avance III HD 500 MHz with 5 mm prodigy H/F-BBO cryoprobe. Native and depolymerized sulfated glycan samples were prepared by dissolving approximately 3.5 mg of each sample in 150 μl of 99.9% D_2_O (CIL). Samples were then transferred to 3 mm NMR tubes for data acquisition. Spectra were acquired at 50 °C by the BCU-I temperature controller unit using 90° experiment with a receiver gain from 69 to 86 for ^1^H spectra and to 189 for the ^1^H-^13^C HSQC, ^1^H-^1^H COSY spectra. The total number of scans used was 256 for ^1^H and 128 for ^1^H-^13^C HSQC and ^1^H-^1^H COSY spectra for the complete acquisition. Postacquisition data processing was performed using zero-filling of the 64K free induction decay data points for ^1^H spectra, 1024 × 128 (F2/F1) for ^1^H-^13^C HSQC spectra, and 2048 × 128 (F2/F1) for ^1^H-^1^H COSY spectra, a Lorentzian-Gaussian window function (exponential factor 1.0 Hz, Gaussian factor 1.0 Hz in GF mode), and used multiple point baseline correction. ^1^H-^13^C HSQC acquisition was performed *via* double Insensitive nuclei enhancement by polarization transfer, including phase sensitivity using Echo/Antiecho-TPPI gradient selection with decoupling during acquisition and using trim pulses in Insensitive nuclei enhancement by polarization transfer with multiplicity editing during the selection step. ^1^H-^1^H COSY acquisition was performed *via* gradient pulses for selection. The acquired spectra were processed using the Mnova NMR software package (v14.2.0, MestreLab Research).

### Structural top-down profiling by HILIC-MS

Top-down analysis was adapted from a previously published method (74). Samples were prepared at a concentration of 10 mg/ml. A Luna HILIC column (2.0 × 150 mm, 200 Å, Phenomenex) was used to separate the intact chains. Mobile phase A was a 5 mM ammonium acetate prepared with HPLC grade water. Mobile phase B was 5 mM ammonium acetate prepared in 98% HPLC grade acetonitrile with 2% of HPLC grade water. An HPLC binary pump was used to deliver the gradient from 10% A to 35% A over 40 min at a flow rate of 150 μl per min. Source parameters for FTMS detection were optimized to minimize in-source fragmentation and sulfate loss and maximize the signal/noise in the negative-ion mode. Optimized parameters, used to prevent in-source fragmentation, included spray voltage of 4.2 kV, capillary voltage of −40 V, tube lens voltage of −100 V, capillary temperature of 275 °C, sheath flow rate of 30 l min^−1^, and auxiliary gas flow rate of 6 l min^−1^. All FT mass spectra were acquired at a resolution of 60,000 with 200 to 2000 Da mass range.

### Bioinformatics for top-down analysis

Charge deconvolution was auto-processed by DeconTools software (75). Structural assignment was done by automatic processing using GlycReSoft software developed at Boston University (76). GlycReSoft parameters were set as Minimum Abundance, 1.0; Minimum Number of Scans, 1; Molecular Weight Lower Boundary, 200 Da; Molecular Weight Upper Boundary, 6000 Da; Mass Shift, ammonium; Match Error (E M), 6.0 ppm; Grouping Error (E G), 80 ppm; Adduct Tolerance (E A), 5. A theoretical database was generated by GlycReSoft.

### Activated partial thromboplastin time

aPTT was performed by incubating 90 μl of plasma with 10 μl of varying concentrations of polysaccharides at 37 °C for 3 min. Hundred microliters of aPTT reagent (# 100402TS, Thermo Fisher Scientific) was then added and the mixture was incubated for 5 min at 37 °C. Clotting time was measured immediately following the addition of 100 μl 25 mM CaCl_2_ (# 100309, Thermo Fisher Scientific). The aPTT readout was measured in seconds. UFH (180 IU/mg) was used as a positive control. The measurements were performed on an Amelung Coagulometer KC4A.

### IIa and Xa inhibition by AT and HCII in the presence of sulfated glycans

Sulfated glycans were assayed for their serpin-mediated inhibitory activity against IIa and Xa using effective concentrations of 10 nM of AT (# HCATIII-0120, Haematologic Technologies) or 25 nM HCII (# HCII-0190, Haematologic Technologies), 2 nM of IIa (# HCT-0020, Haematologic Technologies) or factor Xa (# HCXA-0060, Haematologic Technologies), and 0 to 100 μg/ml of sulfated glycans in 100 μl of TS/PEG buffer (20 mM Tris–HCl, 0.15 M NaCl, and 1.0 mg/ml PEG 8000, pH 7.4) as reported earlier (77). Sulfated glycans (10 μl) at varying concentrations were dispensed into a 96-well microtiter plate, followed by the addition of 40 μl AT (25 nM) or HCII (25 nM). A 50 μl aliquot of IIa (4 nM) or Xa (4 nM) was added last to initiate the reaction. The plate was then immediately incubated at 37 °C for 1 min. This was followed by the addition of 25 μl of chromogenic substrate S-2238 (# S820324, Chromogenix) for IIa or CS–11 (32) (# 229011, Hyphen-BioMed) for factor Xa. Absorbances were then measured at 405 nm for 300 s at an interval of 15 s. Wells without sulfated glycans served as controls and IIa/Xa activity in the control was considered 100%. Residual activity in treated wells was calculated relative to that observed in control wells. Heparin (180 IU/mg) was used in all assays as a positive control.

### Cell and viral cultures

HEK-293T human embryonic kidney cells were purchased from ATCC (CRL-3216). HEK-293T cells expressing human angiotensin-converting enzyme 2 (HEK-293T-hACE2) were purchased from BEI Resources (NR-52511). Both were cultured using Dulbecco’s modified Eagle medium (DMEM) supplemented with 10% heat-inactivated fetal bovine serum, 10,000 IU/l penicillin, 10 mg/l streptomycin, and 29.2 mg/ml L-glutamine (all purchased from Life Technologies). The cells were cultured at 37 °C in a 5% CO_2_ atmosphere.

### Production of pseudotyped lentivirus

Lentivirus particles pseudotyped with SARS-CoV-2 S-protein were prepared as described with minor modifications (78). Briefly, HEK-293T cells were plated to 50% to 70% confluency in six-well plates. Cells were cotransfected with a five-plasmid DNA mixture using BioT transfection reagent (Bioland Scientific) following the manufacturer’s instructions. The five-plasmid DNA mixture added per well consisted of 1 μg pHAGE-CMV-Luc2-IRES-ZsGreen-W (BEI, # NR-52516), 0.22 μg HDM-Hgpm2 (BEI, # NR-52517), 0.22 μg HDM-tat1b (BEI, # NR-52518), 0.22 μg pRC-CMV-Rev1b (BEI,# NR-52519), and 0.34 μg of pGBW-m4137383 (AddGene, # 149541). The latter encodes SARS-CoV-2 S-protein (Wuhan strain) with an 18-amino acid C-terminal truncation. The medium was changed 18 to 24 h posttransfection, and lentivirus stocks were prepared by filtering culture medium collected 60 h posttransfection with a 0.2 μm syringe filter. Stocks were either stored at 4 °C for immediate use or frozen at −80 °C. Titers of lentiviral stocks were determined after a single freeze-thaw by transducing HEK-293T-hACE2 cells with serial dilutions of stocks and counting the resulting numbers of GFP-positive cells 48 h posttransduction.

### GFP-based assay of antiviral activity

Eleven three-fold serial dilutions of heparin, BoSG, or BoSG derivatives were prepared in DMEM. Final concentrations ranged from 400 μg/ml to 6.7 ng/ml. Confluent monolayers of HEK-293T-hACE2 cells in 384-well plates were prepared and treated with compound dilutions in triplicate. After 1 h of incubation, cells were transduced with SARS-CoV-2 S-protein pseudotyped lentivirus. Following incubation for 2 days, relative fluorescence units of GFP fluorescence were quantified using a BioTek Synergy HT Multi-Mode Microplate reader. EC_50_ values were determined as inflection points of four-parameter curves fitted using Prism 5 software (Graphpad) as described (43) and reported as means from three independent experiments. Graphical representations were normalized to % maximum relative fluorescence units.

### Cytotoxicity

Replicate HEK-293T-hACE2 cell cultures were prepared simultaneously with those described above but were not transduced. After incubation of 2 days, cell viability was determined by removing 50 μl of culture media from each well, adding 50 μl of CellTiter-Glo reagent (Promega), incubation for 10 min at room temperature, and measuring relative light units using a BioTek Synergy HT Multi-Mode Microplate reader. 50% cytotoxicity concentrations (TC_50_) were determined as inflection points of four-parameter curves fitted using Prism 5 software (Graphpad) as described (43) and reported as means from three independent experiments. Graphical representations were normalized to % maximum relative light units.

### Preparation of heparin-immobilized SPR sensor chip

Biochip biotinylated heparin was prepared by conjugating its reducing end to amine-PEG3-Biotin (Pierce). In brief, heparin (2 mg) and amine-PEG3-Biotin (2 mg, Pierce) were dissolved in 200 μl H_2_O and 10 mg NaCNBH_3_ was added. The reaction mixture was heated at 70 °C for 24 h. An additional 10 mg NaCNBH_3_ was then added and the reaction was heated at 70 °C for another 24 h. After cooling to room temperature, the mixture was desalted with the spin column (3000 MWCO). Biotinylated heparin was collected, freeze-dried, and used for SA chip preparation. Biotinylated heparin was immobilized to SA chips based on the manufacturer’s protocol. Successful immobilization of heparin was confirmed by the observation of a ∼200 resonance unit (RU) increase on the sensor chip. The control flow cell (FC1) was prepared by 1 min injection with saturated biotin.

Protein samples, including three coagulation factors and four SARS-CoV-2 S-proteins, were diluted in HBS-EP+ buffer (0.01 M Hepes, 0.15 M NaCl, 3 mM EDTA, 0.05% surfactant P20, pH 7.4) at varying protein concentrations and injected at a flow rate of 30 μl/min. At the end of each sample injection, HBS-EP+ buffer was flowed over the sensor surface to facilitate dissociation. After a 3 min dissociation, the sensor surface was regenerated by injecting 30 μl of 2 M NaCl to fully regenerate the surface. Responses were monitored as a function of time (sensorgram) at 25 °C.

### SPR solution competition study of BoSG *versus* heparin

A solution competition study between surface heparin and BoSG in solution to measure IC_50_ was performed using SPR (79). In brief, coagulation (co)factors or S-protein RBDs (250 nM sample) were mixed with different concentrations of BoSG and heparin in HBS-EP buffer and the mixtures injected over a heparin chip at a flow rate of 30 μl/min. After each run, the dissociation and regeneration were performed as described above. For each set of competition experiments, a control experiment (protein only) was performed to make sure the surface was completely regenerated and that the results obtained between runs were comparable. IC_50_ values reflecting the concentration of the competing analyte resulting in a 50% decrease in protein binding (measured in RUs) were calculated from the plots of RUs *versus* concentration of sulfated glycan.

### Generation of 3D structures of BoSG and heparin disaccharides

The initial structures of the BoSG and heparin disaccharides were generated using GLYCAM-Web (glycam.org) (80) and energy-minimized. The structures of the BoSG disaccharides are the following: [β-_D_-Gal-(4SO_3_^−^)-(1→4)-α-_D_-Gal-(3SO_3_^−^)] for [4S-3S], [β-_D_-Gal-(2SO_3_^−^)-(1→4)-α-_D_-Gal-(3SO_3_^−^)] for [2S-3S], [β-_D_-Gal-(4SO_3_^−^)-(1→4)-α-_D_-Gal-(2SO_3_^−^)] for [4S-2S], and [β-_D_-Gal-(2SO_3_^−^)-(1→4)-α-_D_-Gal-(2SO_3_^−^)] for [2S-2S]. The structure of the heparin disaccharide is the following [α-_L_-IdoA-(2SO_3_^−^)-(1→4)-α-_D_-Glc-(N,6-di-SO_3_^−^)].

### Generation of 3D structures of the mutant S-protein RBDs

The 3D structure of the S-protein RBD was obtained from the Protein Data Bank (PDB: 6M0J) (14). The published equilibrated 3D structure of the mutant variant N501Y S-protein RBD (65) was used for molecular docking.

### Molecular docking

Molecular docking of energy-minimized heparin or BoSG disaccharides to the equilibrated 3D structure of N501Y S-protein RBD was performed using AutoDock Vina (81). AutoDockTools was used to retain polar hydrogens, and Gasteiger charges were added to the proteins and glycans (82, 83). Heparin and the BoSG disaccharides were allowed to be flexible during the molecular docking studies. The docking box was 30 × 30 × 30 Å^3^, with the coordinates of the oxygen atom in the side chain of residue Y453 serving as the box center. An exhaustiveness of 5 and a seed value of 0 were used. A fixed seed value in AutoDock Vina makes the predictions deterministic and assures that the same results will be obtained in multiple runs (65). The energy range cut-off was set to 5 kcal∙mol^−1^. For each calculation, 50 docking poses were obtained. The best scored docking pose of the heparin disaccharide and of each galactan, ranked by the AutoDock Vina scoring function, are shown in Figure 6.

## Data availability

All data are contained within this article and will be made available upon request.

## Supporting information

This article contains supporting information.

## Conflict of interest

The authors declare that they have no conflicts of interest with the contents of this article. The funders had no role in the design, writing, or decision of this publication.
