# Supplementary material for: Fractionation of sulfated galactan from the red alga Botryocladia occidentalis separates its anticoagulant and anti-SARS-CoV-2 properties
Source: J Biol Chem. 2022 Mar 23;298(5):101856. doi: 10.1016/j.jbc.2022.101856 (PMC8940257; doi:10.1016/j.jbc.2022.101856)
Supplement: Supplemental Figures S1–S3 [file mmc1.docx]

**SUPPORTING INFORMATION**

**Fractionation of sulfated galactan from the red alga *Botryocladia occidentalis* separates its anticoagulant and anti-SARS-CoV-2 properties**

Seon Beom Kim, Mary Zoepfl, Priyanka Samanta, Fuming Zhang, Ke Xia, Reena Thara, Robert J. Linhardt, Robert J. Doerksen, Michael A. McVoy, Vitor H. Pomin

**Figure S1.** (A) Anion-exchange chromatography on DEAE Sephacel equilibrated with 50 mM sodium acetate. Peaks were obtained using a NaCl linear gradient (dotted line). Fractions were detected by metachromatic property using DMB absorbance at 525 nm (squares). The main fraction containing the BoSG is indicated with a dashed box. (B) 1D ^1^H NMR spectrum of BoSG after chromatography (δ_H_ expansion from 5.9 to 2.9 ppm) showing similar ^1^H signal profile observed in previous references. Spectrum was recorded in D_2_O at 50 ^o^C on a Bruker 500 MHz instrument.

^
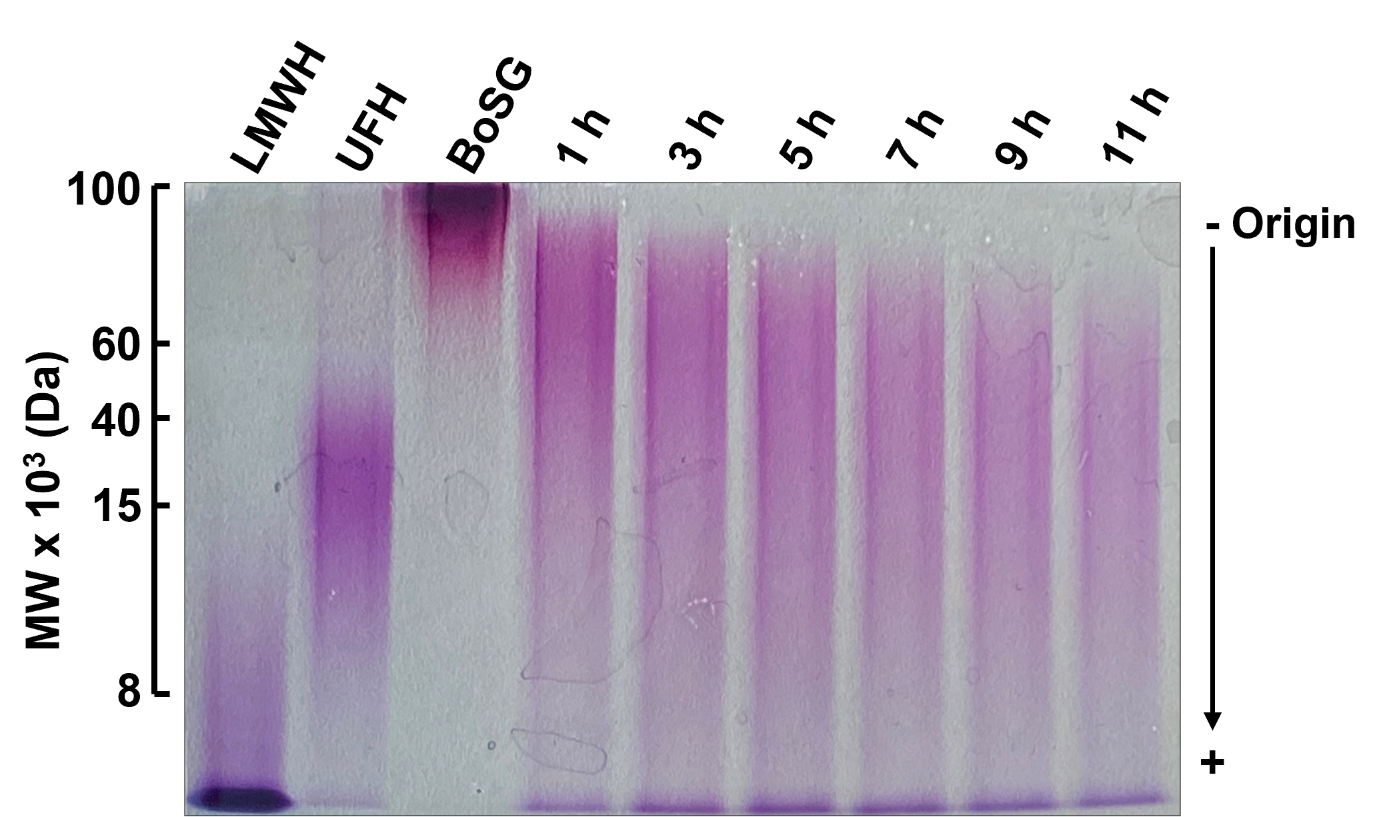
^

**Figure S2.** Electrophoretic mobility of BoSG and hydrolyzed derivatives obtained after incubation in 0.1 M HCl at 60 ℃ for 1, 3, 5, 7, 9 or 11 h. Samples were separated on a 22% polyacrylamide gel and stained with toluidine blue. Molecular weight markers include low molecular weight heparin (LMWH, ~ 8 kDa) and unfractionated heparin (UFH, ~ 15 kDa). The majority of native BoSG has molecular weight above 100 kDa and does not migrate into the gel.

**
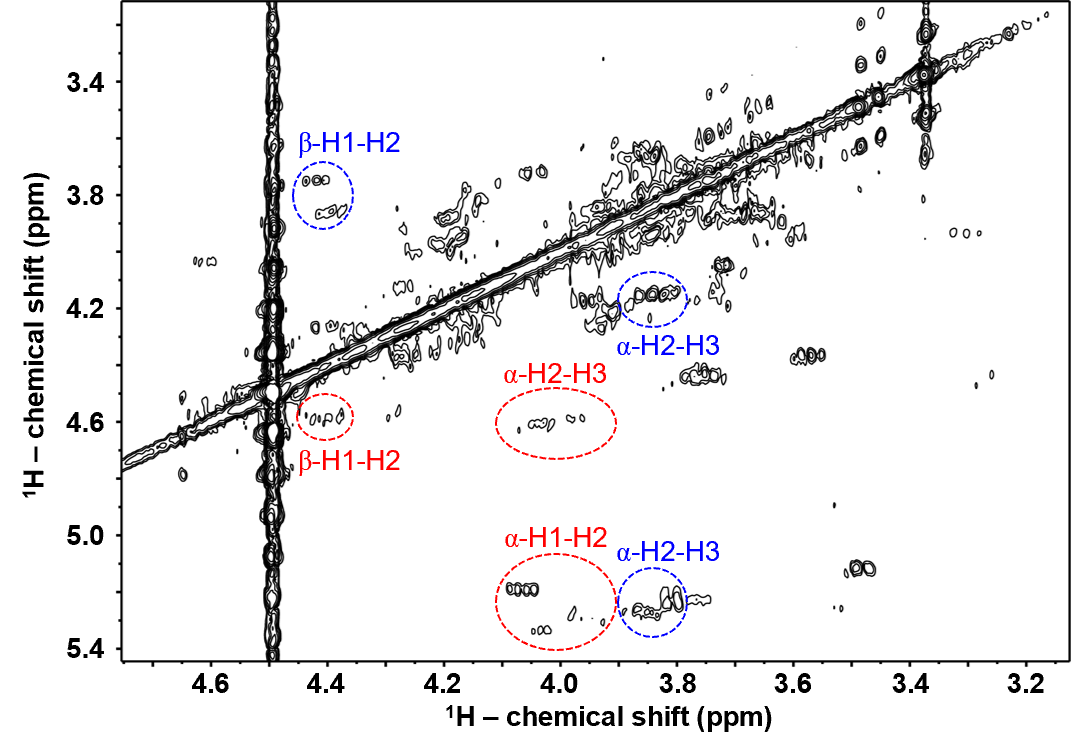
**

**Figure S3.** 2D ^1^H-^1^H COSY spectra of Fr4 were recorded in D_2_O at 50 ℃ on a 500 MHz Bruker NMR instrument. The ^1^H-^1^H cross peaks of sulfated (red) and non-sulfated (blue) α and β unit are labeled in the 2D cross-peaks using Greek letters denoting the anomeric (ring) unit followed by a number of the ^1^H-^1^H pair of the galactose ring. The correlation of H1-H2 and H2-H3 peaks are indicated in the COSY spectrum with dashed circles.
